# Application of Quantitative Parameters of Contrast-Enhanced Ultrasound in Common Benign and Malignant Lesions in Pediatric Livers: A Preliminary Study

**DOI:** 10.3390/diagnostics13223443

**Published:** 2023-11-14

**Authors:** Dan Han, Ting Wang, Ruiqi Wang, Jingyu Chen, Yi Tang

**Affiliations:** Department of Ultrasound, Children’s Hospital of Chongqing Medical University, National Clinical Research Center for Child Health and Disorders, Ministry of Education Key Laboratory of Child Development and Disorders, Chongqing Key Laboratory of Pediatrics, Chongqing 400014, China; 2021120616@stu.cqmu.edu.cn (D.H.); 484693@hospital.cqmu.edu.cn (T.W.); 2022120643@stu.cqmu.edu.cn (R.W.); cjy419103@163.com (J.C.)

**Keywords:** contrast-enhanced ultrasound, time–intensity curve, quantitative analysis, child, benign and malignant lesions of the liver, accuracy

## Abstract

We aimed to investigate the diagnostic utility of quantitative parameters of contrast-enhanced ultrasound (CEUS) for benign and malignant liver lesions in pediatric patients. This was a single-center retrospective analysis of children with liver lesions who underwent CEUS at our hospital between July 2019 and February 2023. The CEUS perfusion patterns for all lesions were qualitatively analyzed using histopathology, contrast-enhanced magnetic resonance imaging, contrast-enhanced computed tomography, or long-term clinical follow-up as reference standards. The CEUS images were quantitatively analyzed using SonoLiver^®^ software (TomTec Imaging Systems, Munich, Germany) to obtain data regarding quantitative parameters and dynamic vascular pattern (DVP) parametric images, including rise time (RT), time to peak (TTP), mean transit time (mTT), and maximum intensity (IMAX). Statistical analysis was carried out using Student’s *t*-test and receiver operating characteristic (ROC) curve analysis to evaluate the diagnostic value of quantitative parameters. A total of 53 pediatric cases were included in this study, and 88.57% (31/35) of malignant lesions exhibited hyper-enhancement with rapid washout patterns; the same proportion of DVP parametric images exhibited washout patterns. Conversely, 94.44% (17/18) of benign lesions showed hyper-enhancement with slow washout patterns, and the same proportion of DVP parametric images showed no-washout patterns. RT, TTP, and mTT were significantly shorter in the malignant group than in the benign group (*p* < 0.05), while IMAX showed no significant difference (*p* > 0.05). ROC analysis indicated that mTT < 113.34 had the highest diagnostic value, with an area under the curve of 0.82. CEUS quantitative analysis had an accuracy of 98.11%, while qualitative analysis had an accuracy of 92.45%, with no statistically significant difference (*p* > 0.05). Quantitative analysis of CEUS provides valuable assistance in differentiating benign and malignant liver lesions in children. Among all quantitative parameters, mTT holds promise as a potentially valuable tool for identifying liver tumors.

## 1. Introduction

Focal liver lesions (FLLs) occur commonly in the pediatric population. The most prevalent lesions are hepatic hemangiomas and hepatoblastomas, with the incidence of hepatoblastoma peaking in children younger than 5 years of age. If treated appropriately, the overall 5-year survival rate of children can reach 80% [1]. Therefore, accurate preoperative diagnosis is crucial in clinical treatment and prognosis assessment. In recent years, various imaging methods have been used to diagnose neoplastic lesions of the liver in children, including contrast-enhanced ultrasound (CEUS). CEUS is a radiation-free imaging modality that diagnoses liver diseases by observing the perfusion characteristics of intralesional contrast agents in the arterial, portal, and delayed phases, and is known to have high safety and sensitivity [2,3]. In 2016, the Food and Drug Administration (FDA) approved use of the ultrasound contrast agent SonoVue in pediatric liver examinations [4]. This provided theoretical and technical support for global pediatric CEUS technology and increased its utilization. Numerous studies have shown that CEUS can be used to characterize focal nodular liver lesions in children [5,6,7]. However, the visual evaluation of contrast ultrasound images is subjective and largely dependent on the observer’s experience, which may lead to biases in the observation and interpretation.

Both primary and metastatic lesions of liver malignancies show hypo-enhancement in the delayed phase. However, this may also occur in some benign lesions, making them challenging to distinguish [8]. The CEUS quantitative software, SonoLiver^®^ (TomTec 1.1, Munich, Germany), can clarify the quantitative analysis of intratumor contrast agent kinetics such that the changes in tumor blood vessels can be represented by color; this helps in objectively displaying the perfusion information of each lesion and overcomes the limitations of simple qualitative assessment [9]. For example, in a retrospective study of 134 adult patients by Schwarz et al. [8], quantitative parameters such as time to ascent and the ratio of lesions to normal liver parenchyma were evaluated. The study demonstrated that quantifying CEUS parameters can help distinguish between malignant and benign liver lesions. Zhang et al. [10] published an article on the quantitative analysis of adult orbital mass lesions based on the time–intensity curve (TIC) in 2021. The study showed a sensitivity of 66.67% for visual assessment and 89.19% for quantitative analysis, significantly improving the accuracy of CEUS and obtaining the optimal cut-off values for parameters to identify benign and malignant lesions in the orbit. Gatos et al. [11] also reported a quantitative TIC analysis, with good diagnostic accuracy of 90.3%. There is limited literature available regarding the quantitative analysis of benign and malignant lesions in pediatric liver cases.

This study aimed to retrospectively analyze CEUS images of liver lesions in pediatric patients and assess the value of quantitative parameters and dynamic vascular pattern (DVP) parametric images in the differential diagnosis of benign and malignant lesions in the liver.

## 2. Materials and Methods

### 2.1. Study Design

The ethics committee of the Children’s Hospital Affiliated to Chongqing Medical University reviewed and approved this study (approval no.: CHCMU-XJS-2019-20). Written informed consent was obtained from all guardians of the children prior to CEUS examination.

### 2.2. Patients

We conducted a retrospective analysis of the CEUS database between July 2019 and February 2023 at our hospital, with reference standards including histopathology, contrast-enhanced magnetic resonance imaging (CEMRI), contrast-enhanced computed tomography (CECT), or long-term clinical follow-up.

**Figure 1:**
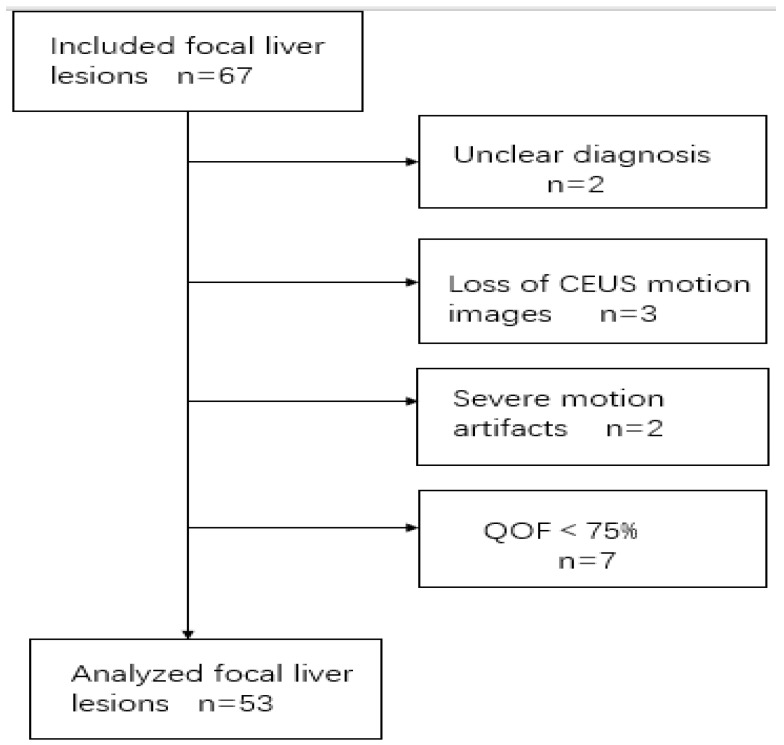
Flowchart of the study population and exclusion criteria.

The inclusion criteria were as follows: (1) <18 years of age, (2) presence of a space-occupying lesion in the liver, and (3) availability of complete clinical data and CEUS images. The exclusion criteria were as follows: (1) unclear final diagnosis, (2) missing dynamic image, (3) presence of severe motion artifacts during post-processing, and (4) CEUS image analysis quality of fit <75%. Figure 1 shows the enrollment of patients according to these criteria.

### 2.3. CEUS and Contrast Agent

Ultrasound examinations were performed by an ARIETTA 70 unit (Hitachi Medical Systems, Tokyo, Japan) equipped with a 1.0–4.0 MHz C251 convex array transducer, or an Acuson Sequoia unit (Siemens Medical Solutions USA, Mountain View, CA, USA) equipped with a 1.0–4.0 MHz 5C1 convex array transducer. These devices were used in conjunction with the SonoLiversoftware. The mechanical index was <0.1. The contrast agent used was SonoVue sulfur hexafluoride gas microbubbles (Bracco, Milano, Italy). The average diameter of the microbubbles was 1.5–2.5 µm, consistent with the size of circulating red blood cells; hence, the bubbles could be exhaled through the lungs after 20 min of injection [12,13]. The FDA-recommended pediatric application dose of 0.03 mL/kg was used [13,14,15]. If the contrast effect based on the body weight ratio was unsatisfactory, the dose was adjusted accordingly to a maximum dose of 2.4 mL per injection.

### 2.4. Imaging and Data Analysis

The liver lesions were first evaluated using conventional ultrasound examination. Then, the ultrasound was switched to dual-screen mode, with the target liver lesions placed at the center of the screen. As per standard protocol, a fixed amount (≤2.4 mL) of contrast agent was injected into the antecubital vein and flushed with 5 mL of saline. A timer was started immediately after the injection. The CEUS data were recorded for 3–6 min and digitally stored on the hard disk. The whole contrast process was divided into the arterial (10–45 s), portal (30–120 s), and delayed phases (>120 s) [6,8,15]. All tests were performed by one of two sonographers with more than 10 years of experience in liver ultrasonography. The same two sonographers reviewed and qualitatively analyzed all the tests to determine by consensus whether the liver lesions were benign or malignant. The time interval between performance and analysis was at least 60 min for each test. The main criterion for malignant liver lesions was a loss of contrast enhancement or a washout starting from the portal phase to the delayed phase. In contrast, benign lesions did not show a washout until the delayed phase [16,17].

### 2.5. Quantitative Analysis

CEUS images were analyzed offline using SonoLiver^®^ software. Three regions of interest (ROIs) were drawn in the panel of the offline software: a blue ROI designated the area of motion compensation, yellow ROI (reference area) delineated the same depth as the normal liver parenchyma, and a green ROI (target lesion area) outlined the area of interest in the lesion. The target lesion area was drawn according to the size of the tumor and contained as much of the lesion as possible while avoiding necrosis, liquefaction, and calcification. Fine adjustments were made to correct respiratory movements and obtain quantitative and DVP parametric images with the reference and target lesion area outputs. These were stored on the hard disk. Quantitative analysis was performed by a sonographer with 2 years of experience who was blinded to the pathological and clinical diagnosis.

The SonoLiver^®^ software was used to obtain perfusion-related TIC for analysis of quantitative perfusion characteristics of blood within tumors and identification of objective quantitative parameters. These parameters included maximum intensity (IMAX), defined as the highest percentage of intensity throughout the perfusion process; rise time (RT), defined as the time from 10% to 90% IMAX; time to peak (TTP), defined as the time when the contrast agent begins to reach the lesion and reaches IMAX; mean transit time (mTT), defined as the time from the start of the contrast clearance to 50% rinse; and quality of fit defined as the correspondence between the raw data and the theoretical curve.

**Figure 2:**
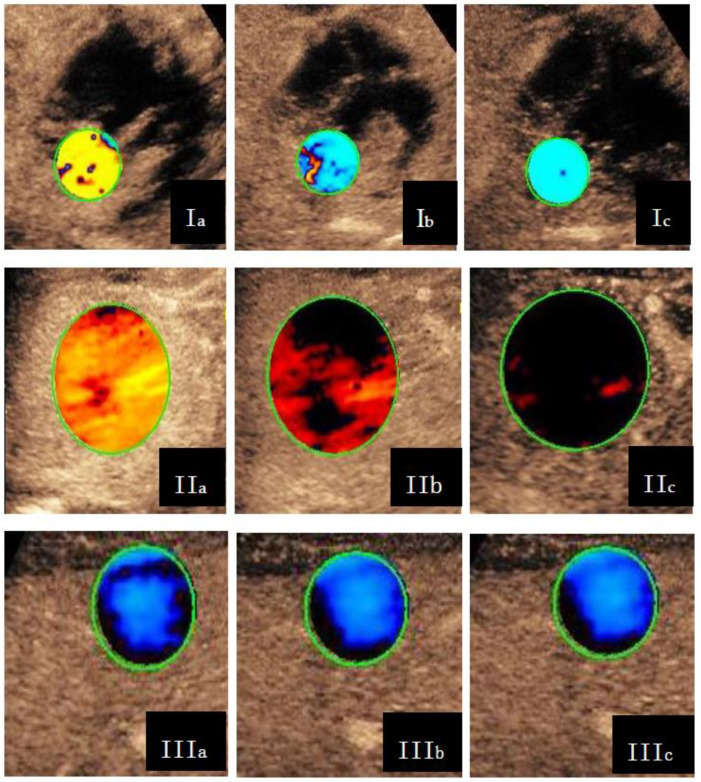
Three types of DVP parametric images. Type I, washout pattern is shown in yellow (**Ia**), green (**Ib**), and green (**Ic**); Type II, no-washout pattern is shown in red (**IIa**), reddish-black (**IIb**), and black (**IIc**); Type III, cystic appears in blue (**IIIa**), blue (**IIIb**), and blue (**IIIc**).

Dynamic vascular pattern curve (DVPC), which is derived from the TIC of the tumor minus that of the surrounding tissue, was used to indicate the difference in enhancement strength between the tumor and the surrounding soft tissue. According to the relationship between the direction of DVPC and the *x*-axis, DVPC is divided into four types: positive and negative biphasic waves, positive wave, negative and positive biphasic waves, and negative wave. DVP parametric images (color-coded images) were automatically generated by the SonoLiver^®^ software: red/yellow (warm color) represents hyper-enhancement, blue/green (cool color) indicates hypo-enhancement, and black indicates iso-enhancement. Depending on the color difference, parametric images were classified into three types: Type I, washout pattern (yellow/red first, blue/green last); Type II, no-washout pattern (always yellow/red or last black); and Type III, cystic (always blue/green) [10,18] (Figure 2).

### 2.6. Statistical Analysis

Statistical analysis was performed using SPSS25.0 statistical software, and the categorical data are expressed as a percentage or ratio, indicated as *n* (%). Age did not conform to the normal distribution and is denoted by *M* (*P*_25_, *P*_75_). All quantitative parameters were compared using an independent sample Student’s *t*-test, and results are expressed as mean ± standard deviation. The chi-square test was used to compare categorical variables. *p* < 0.05 was considered statistically significant. All parameters were further analyzed using the receiver operating characteristic (ROC) curve to obtain a suitable threshold for diagnosis; area under the curve (AUC), 95% confidence interval (CI), sensitivity, and specificity were measured.

## 3. Results

A total of 53 pediatric cases were included in this study (Table 1). Within this cohort, there were 26 cases of hepatoblastoma, 8 cases of hepatic hemangioma, 6 cases of liver metastases, 10 cases of focal nodular hyperplasia of the liver, and 3 cases of hepatocellular carcinoma. Regarding patient characteristics, there were 24 male and 29 female patients, whose ages ranged from 0.53 to 144 months (average age: 26.00 [9.10, 61.00] months). The lesion sizes varied from 0.8 to 20.0 cm, with 35 cases categorized in the malignant group and 18 in the benign group. Among these cases, 43 were confirmed by pathology, 8 by CEMRI or CECT, and 2 by long-term clinical follow-up.

Among primary hepatoblastomas, 92.31% (24/26) showed portal phase hypo-enhancement and 7.69% (2/26) showed iso-enhancement. All hepatocellular carcinomas exhibited rapid enhancement in the arterial phase and hypo-enhancement in the portal and delayed phases. Certain lesions exhibited peculiar perfusion patterns. For instance, 66.67% (4/6) of liver metastases displayed hyper-enhancement during the arterial phase, followed by early washout, with portal and delayed phases demonstrating “black hole” appearances. Further, 87.5% (7/8) of hemangiomas presented with periarterial nodular and centripetal perfusion enhancement, accompanied by hyper-enhancement or iso-enhancement during the portal and delayed phases. In the early arterial phase, 70% (7/10) of the focal nodular hyperplasia cases showed enhanced spoke-wheel-shaped and centrifugal behaviors. Table 2 presents these findings.

**Figure 3:**
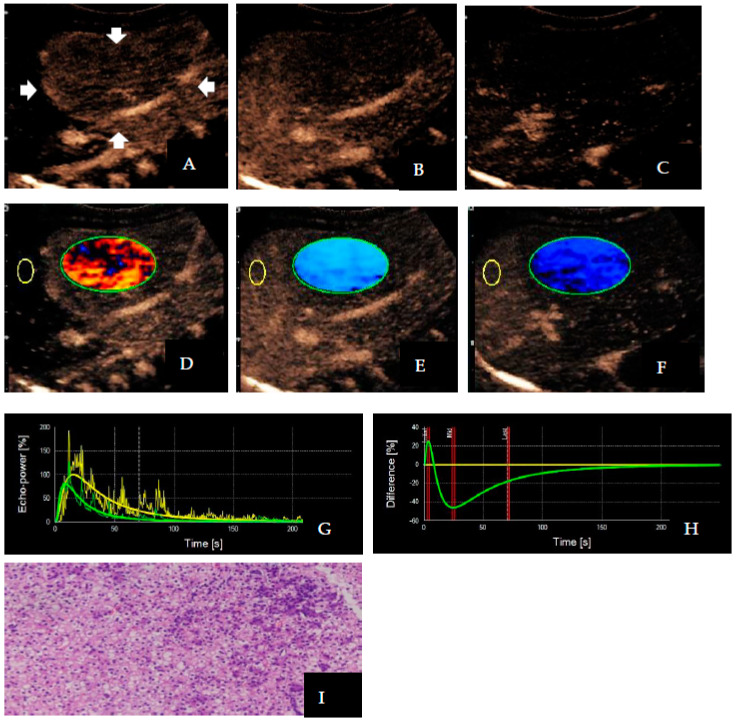
Images of a 4-month-old boy with confirmed hepatoblastoma. The lesion is located in the right lobe of the liver (arrows). Contrast-enhanced ultrasound enhancement images showed (**A**) hyper-enhancement (5 s), (**B**) hypo-enhancement (25 s), and sustained (**C**) hypo-enhancement (70 s); as a result, dynamic vascular pattern parametric images appeared (**D**) red, (**E**) blue, and (**F**) blue, respectively (Type I). The effect of the portal washout on the parametric images was evident, despite the subtle effect of the washout in the portal phase on contrast-enhanced ultrasound. The time–intensity curve showed (**G**) rapidly increasing and rapidly decreasing waves, and the dynamic vascular pattern showed positive and negative biphasic waves (**H**); the puncture specimen microscopically showed (**I**) epithelioid tumor cells, with focal nuclei of large, dark staining in a nested cluster, diagnosed as hepatoblastoma (HE × 40). In this case, the yellow circle represents the region of interest in the reference area and the green circle represents the region of interest in the target lesion area; the green curve indicates the enhancement of the lesion, the yellow curve indicates the enhancement of the adjacent hepatic parenchyma, and the yellow curve flattens out in the dynamic vascular pattern curve. The three red lines in (**H**) represent three different time points where the contrast agent enters.

**Figure 4:**
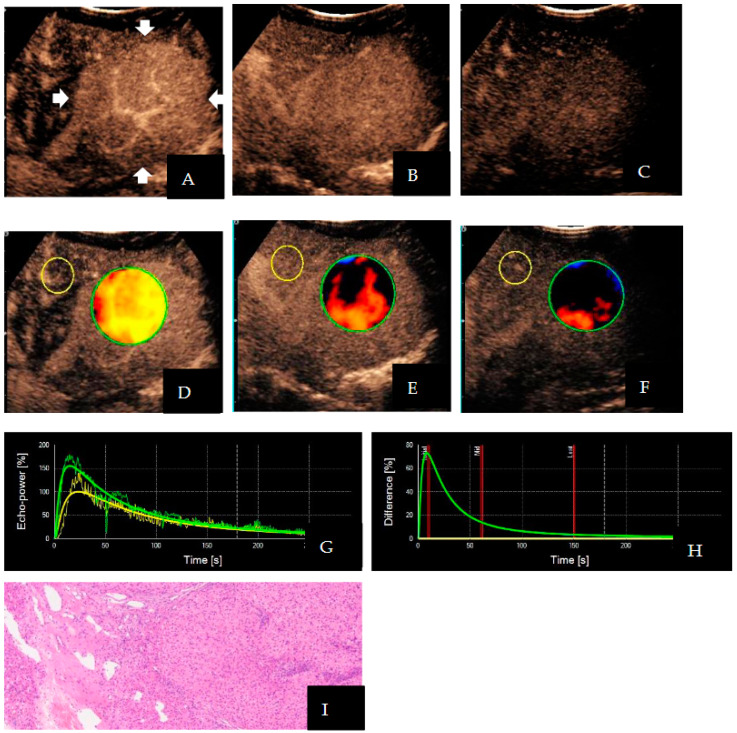
Images of a 9-year-old girl with confirmed focal nodular hyperplasia. The lesion is located in the left lobe of the liver (arrows). Contrast-enhanced ultrasound enhancement images showed (**A**) hyper-enhancement (20 s), (**B**) slight hyper-enhancement (60 s), and (**C**) iso-enhancement (150 s); as a result, dynamic vascular pattern parametric images appeared (**D**) yellow, (**E**) reddish-black, and (**F**) black, respectively (Type II). Although the visual analysis the slight hyper-enhancement was subjective on contrast-enhanced ultrasound, it was objective on parametric images. The time–intensity curve showed (**G**) rapidly increasing and slowly decreasing waves, and the dynamic vascular pattern showed (**H**) positive waves; the puncture specimen microscopically showed (**I**) nodular proliferation of hepatocytes surrounded by fibrous tissues, diagnosed as focal nodular hyperplasia (HE × 40). In this case, the yellow circle represents the region of interest in the reference area and the green circle represents the region of interest in the target lesion area; the green curve indicates the enhancement of the lesion, the yellow curve indicates the enhancement of the adjacent hepatic parenchyma, and the yellow curve flattens out in the dynamic vascular pattern curve. The three red lines in (**H**) represent three different time points where the contrast agent enters.

The distribution of TIC and DVP differed significantly between benign and malignant liver tumors (Table 3 and Table 4). In 88.57% (31/35) of cases involving malignant liver lesions, hyper-enhancement with rapid washout patterns was observed. The TIC showed rapidly increasing and rapidly decreasing waves, and the DVP showed positive and negative biphasic waves (Figure 3). However, 94.44% (17/18) of benign liver lesions showed hyper-enhancement with slow washout patterns, TIC showed rapidly increasing and slowly decreasing waves, and DVP showed positive wave (Figure 4). Only one malignant tumor with sacrococcygeal yolk cystoma liver metastasis showed hypo-enhancement with rapid washout patterns. The DVP displayed a negative wave in this case. For DVP parametric images, the percentages of Types I, II, and III were 60.38% (32/53), 37.73% (20/53), and 1.89% (1/53), respectively. Among them, Type I accounted for 88.57% (31/35) of the malignant liver tumors, and Type II accounted for 94.44% (17/18) of the benign liver tumors.

Quantitative analysis based on TIC showed that the RT, TTP, and mTT of the malignant group were shorter than those of the benign group (9.93 ± 4.37 s vs. 14.12 ± 5.51 s, 11.37 ± 4.57 s vs. 14.97 ± 5.53 s, and 57.31 ± 51.08 s vs. 188.88 ± 170.70 s, respectively), and the difference was statistically significant (*p* = 0.004, 0.015, 0.005, respectively). However, the IMAX showed no statistically significant difference between the two groups (267.77 ± 292.32% vs. 240.20 ± 172.54%, *p* = 0.715) (Table 5).

**Figure 5:**
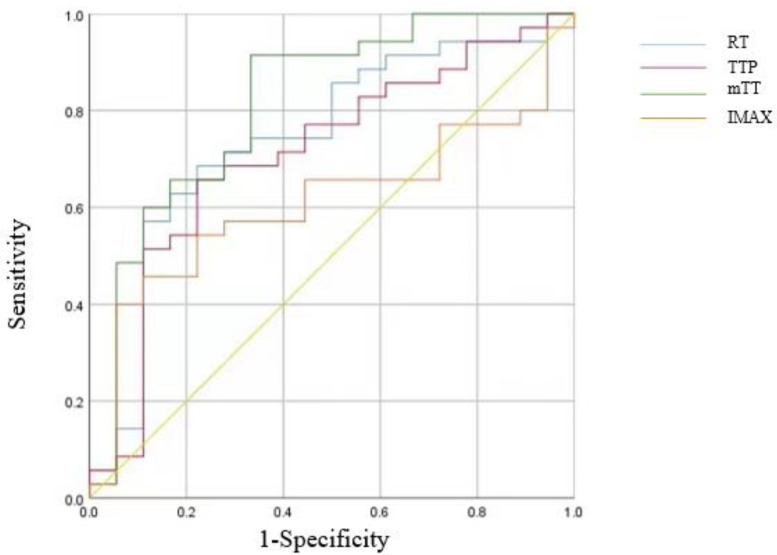
ROC curve of TIC quantitative parameters.

ROC curve analysis showed that the best cut-off values for identifying malignant tumors were RT = 11.16, TTP = 11.42, and mTT = 113.34. Among these three parameters, mTT < 113.34 was the most diagnostic, with an AUC of 0.82, 95% CI of 68.6–94.6%, sensitivity of 91.4%, and specificity of 66.7% (Figure 5 and Table 6).

Combining quantitative parameters and DVP parametric images in CEUS quantitative analysis for diagnosing benign and malignant liver lesions in pediatric patients yielded a sensitivity of 100.00%, specificity of 94.44%, diagnostic accuracy of 98.11%, positive predictive value of 97.22%, and negative predictive value of 100.00%. In contrast, qualitative analysis resulted in a sensitivity of 91.43%, specificity of 94.44%, diagnostic accuracy of 92.45%, positive predictive value of 96.97%, and negative predictive value of 85.00%. No significant differences were observed between the two methods (*p* > 0.05) (Table 7).

## 4. Discussion

CEUS imaging plays an important role in evaluating FLLs. In a pediatric liver, the pattern of enhancement of malignant lesions and the perfusion of blood flow differ significantly from those of benign lesions. Most malignant lesions typically present as irregular hyper-enhancement compared with adjacent liver parenchymal lesions. In this study, 91.43% (32/35) of cases showed hyper-enhancement during the portal or delayed phase, while 94.44% (17/18) of benign lesions exhibited sustained enhancement, consistent with findings of prior studies [3,19]. In this study, the quantitative analysis software, SonoLiver^®^, was used to improve the diagnostic accuracy of suspicious liver lesions. This software enables linearization of video data and generates and displays TIC in the form of parametric images. The integration of dynamic vascular features into a single image reflects the distribution of hemoperfusion in the lesion [20]. Therefore, DVP parametric images could clearly display enhanced rapid changes and details, especially subtle features that might be challenging to discern in qualitative analysis. Time-related information of tumors, as well as contrast enhancement, can be visually presented in the parametric image, which greatly improves the visualization of CEUS images. Most pediatric malignant liver lesions showed a washout pattern in the DVP parametric images, while benign lesions predominantly exhibited a no-washout pattern. The cystic type may be attributed to tumor necrosis or persistent insufficient enhancement.

In quantitative analysis, parameters such as RT and TTP are related to contrast filling velocity, mTT is associated with contrast washout speed, and IMAX can provide information regarding lesion blood volume and flow related to perfusion [21,22]. The three time-related parameters (RT, TTP, and mTT) showed significant differences between benign and malignant liver lesions, with the malignant group having significantly lower RT, TTP, and mTT values. In contrast, the volume-related parameter (IMAX) did not differ significantly between the two groups. Among these parameters, mTT < 113.34 had the highest diagnostic value, with high AUC, 95% CI, and sensitivity. The decreased mTT could be attributed to the disordered vessel formation within malignant tumors, potentially caused by the absence of a portal blood supply and the disruption of a typical venous return system. This disruption of tumor vessels results in arteriovenous shunting and fistula formation, allowing SonoVue microbubbles to pass through malignant lesions more quickly. In contrast, benign tumors, such as hemangiomas, are primarily composed of blood sinusoids, leading to slower blood flow and delayed SonoVue washout. Therefore, mTT was significantly reduced in malignant tumors compared with that in benign liver focal lesions, consistent with the results of Zheng et al. [18]. Only a few benign lesions had mTT values lower than those of malignant lesions, especially when hemangiomas were suspected as malignant tumors. In this study, certain hemangiomas with larger diameters exhibited arteriovenous fistulas, presenting as incomplete filling with a minor hypo-enhancement during both the portal and delayed phases. This phenomenon was effectively detected by the quantitative analysis software, resulting in a calculated mTT < 113.34. However, the associated DVP parametric images still displayed a no-washout pattern. Consequently, this cannot be attributed to a typical washout phenomenon as these hemangiomas did not exhibit significant hypo-enhancement over time, consistent with the results of Schwarz et al. [8]. These findings indicate that mTT < 113.34 may introduce errors in the diagnosis of malignant liver lesions. However, combining quantitative analysis with DVP parametric images reduced error rates. To enhance accuracy, we recommend a combination of quantitative parameters and DVP parametric images to differentiate between benign and malignant liver tumors.

IMAX, a volume-related parameter, was not significantly different between the benign and malignant tumors. This may be due to the relatively small sample size in this single-center study. Therefore, a larger sample size and multi-center studies are necessary to determine the diagnostic significance of IMAX for benign and malignant liver tumors in pediatric patients.

CEUS was observed to significantly improve the diagnosis of FLLs. In this study, two sonographers with over 10 years of experience in liver diagnosis achieved high accuracy in qualitative diagnosis. However, qualitative diagnosis largely depends on the observer’s experience. Quaia et al. [23] reported moderate consistency between sonographers with different years of experience; kappa values for qualitative diagnosis ranged from 0.47 to 0.63. In contrast, quantitative analysis, as reported by Anay et al. [24], demonstrated excellent consistency among sonographers, with the kappa values increasing from 0.54 in qualitative analysis to 0.99 in quantitative analysis. In summary, quantitative analysis using CEUS has high consistency in differential diagnosis among experienced observers. Through objectivity and quantification, CEUS quantitative data can also provide new possibilities for future deep learning to achieve more accurate diagnosis and treatment [25,26].

## 5. Limitations

This study has several limitations. First, this was a single-center retrospective study; the number of cases was relatively small, the number of lesions in each category was uneven, the age distribution was uneven, and there was a lack of cases in the 12–18-year age group. Second, the sampling depth in the ROI and reference region was different because of the large tumor size in some children. Third, parametric imaging may be affected by respiratory movements or heartbeat, even when motion compensation is applied.

## 6. Conclusions

The TIC of CEUS can provide quantitative parameters in the ROI, enabling precise differentiation in terms of the time of contrast agent entry, peak concentration, and washout time. This refinement enhances the diagnostic accuracy of CEUS, particularly when assessing liver lesions in pediatric cases, offering valuable insights for distinguishing between benign and malignant lesions. Among all quantitative parameters, mTT holds promise as a potentially valuable tool for identifying liver tumors.

## Figures and Tables

**Table 1:** General information of children with benign and malignant lesions of the liver.

Tumor Type	Hepatoblastoma	Hepatic Metastases	Hepatocellular Carcinoma	Hemangioma	Focal Nodular Hyperplasia
All patients (*n* = 53)	26	6	3	8	10
Age (months)	1.6~124	6.93~144	96~144	0.53~96	24~127
Gender					
Male	12	4	1	6	1
Female	14	2	2	2	9
Tumor diameter (cm)	2.4~16.0	0.8~4.4	9.1~20.0	2.0~12.8	3.5~7.6
Histopathology	23	6	3	2	9
CECT/CEMRI	3	0	0	5	0
Long-term clinical follow-up	0	0	0	1	1

**Table 2:** The enhanced level of contrast-enhanced ultrasound for liver tumors.

Tumor Type	All Patients	Arterial Phase			Portal Phase			Delayed Phase		
		Hyper	Echo	Hypo	Hyper	Echo	Hypo	Hyper	Echo	Hypo
Hepatoblastoma	26	26	0	0	1	1	24	0	2	24
Hepatic metastases	6	5	0	1	1	0	5	0	1	5
Hepatocellular carcinoma	3	3	0	0	0	0	3	0	0	3
Hemangioma	8	8	0	0	5	2	1	2	5	1
Focal nodular hyperplasia	10	10	0	0	9	1	0	3	7	0

**Table 3:** TIC type comparison.

Group	Patients (*n* = 53)	Rapidly Increasing and Rapidly Decreasing Waves	Rapidly Increasing and Slowly Decreasing Waves	Slowly Increasing and Slowly Decreasing Waves	Slowly Increasing and Rapidly Decreasing Waves
Benign	18	1	17		0
	(33.96%)	(5.56%)	(94.44%)	0	
Malignant	35	31	3		1
	(66.04%)	(88.57%)	(8.57%)	0	(2.86%)
Total	53	32	20		1
	(100.00%)	(60.38%)	(37.73%)	0	(1.89%)
*p* Value	0.000				

**Table 4:** DVPC type comparison.

Group	Patients (*n* = 53)	Positive and Negative Biphasic Waves	Positive Wave	Negative and Positive Biphasic Waves	Negative Wave
Benign	18	1	17		0
	(33.96%)	(5.56%)	(94.44%)	0	
Malignancy	35	31	3		1
	(66.04%)	(88.57%)	(8.57%)	0	(2.86%)
Total	53	32	20		1
	(100.00%)	(60.38%)	(37.73%)	0	(1.89%)
*p* Value	0.000				

**Table 5:** Malignant and benign tumor quantitative parameters.

Group	RT (s)	TTP (s)	mTT (s)	IMAX (%)
Benign group	14.12 ± 5.51	14.97 ± 5.53	188.88 ± 170.70	240.20 ± 172.54
Malignant group	9.93 ± 4.37	11.37 ± 4.57	57.31 ± 51.08	267.77 ± 292.32
*t* Value	3.026	2.521	3.197	−0.367
*p* Value	0.004	0.015	0.005	0.715

**Table 6:** ROC curve analysis of quantitative parameters in the liver tumors.

Parameters	AUC	95%CI	Cutoff Value	Sensitivity	Specificity	*p* Value
RT	0.743	0.596–0.889	11.16	0.686	0.778	0.004
TTP	0.710	0.558–0.861	11.42	0.657	0.778	0.013
mTT	0.816	0.686–0.946	113.34	0.914	0.667	0.000
IMAX	0.610	0.456–0.763	132.21	0.457	0.889	0.195

**Table 7:** Diagnostic results of quantitative and qualitative analysis of CEUS for the differentiation of benign and malignant hepatic lesions in children (%).

Method	Quantitative Analysis	Qualitative Analysis	*χ* 2Value	*p* Value
Patients (*n* = 53)	53	53		
Sensitivity	100.00 (35/35)	91.43 (32/35)	0.069	0.793
Specificity	94.44 (17/18)	94.44 (17/18)	0.000	1.000
Accuracy	98.11 (52/53)	92.45 (49/53)	0.046	0.830
PPV	97.22 (35/36)	96.97 (32/33)	0.009	0.924
NPV	100.00 (17/17)	85.00 (17/20)	0.117	0.732

Note. PPV: positive predictive value; NPV: negative predictive value.

## Data Availability

The data are included in the article.
